# Supplementary material for: Deciphering the mechanism of processive ssDNA digestion by the Dna2-RPA ensemble
Source: Nat Commun. 2022 Jan 18;13:359. doi: 10.1038/s41467-021-27940-y (PMC8766458; doi:10.1038/s41467-021-27940-y)
Supplement: Supplementary file 1 — Supplementary Information [file 41467_2021_27940_MOESM1_ESM.pdf]

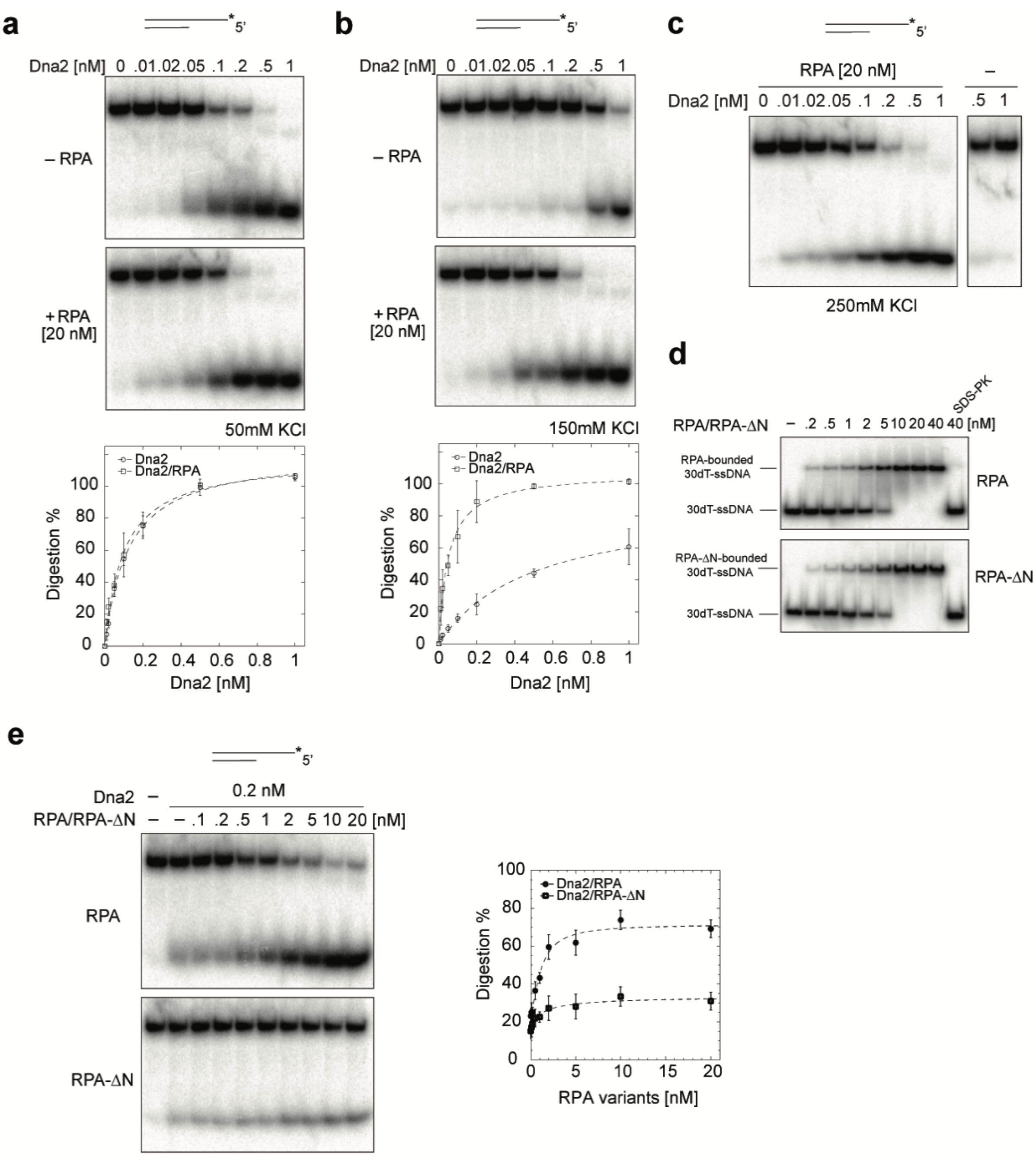

Supplementary Figure 1. Digestion of 5’-labeled short overhanging DNA by Dna2-RPA ensemble: salt dependency and the function of RPA-N. a-c. Digestion by titrated Dna2 (0 nM to 1 nM) on 5’-labeled 40-nt 5’-overhanging DNA (5 nM) without and with RPA (20 nM) at 50 mM KCl (a), 150 mM KCl (b) and 250 mM (c). The experiments were repeated three times. For quantification, mean values ± s.d. from three independent experiments were plotted for a-b. d. Comparison of ssDNA binding affinities of titrated RPA and RPA-ΔN (0 nM to 40 nM) on 5’-labeled $dT_{(30)}$ ssDNA (5 nM) at 150 mM KCl and the experiments were repeated three times. e. Impact of titrated RPA and RPA-ΔN (0 nM to 20 nM), on the digestion of 5’-labeled 40-nt 5’-overhanging ssDNA (5 nM) by Dna2 (0.2 nM). For quantification, mean values ± s.d. from three independent experiments were plotted. Source data are provided as a Source Data file.

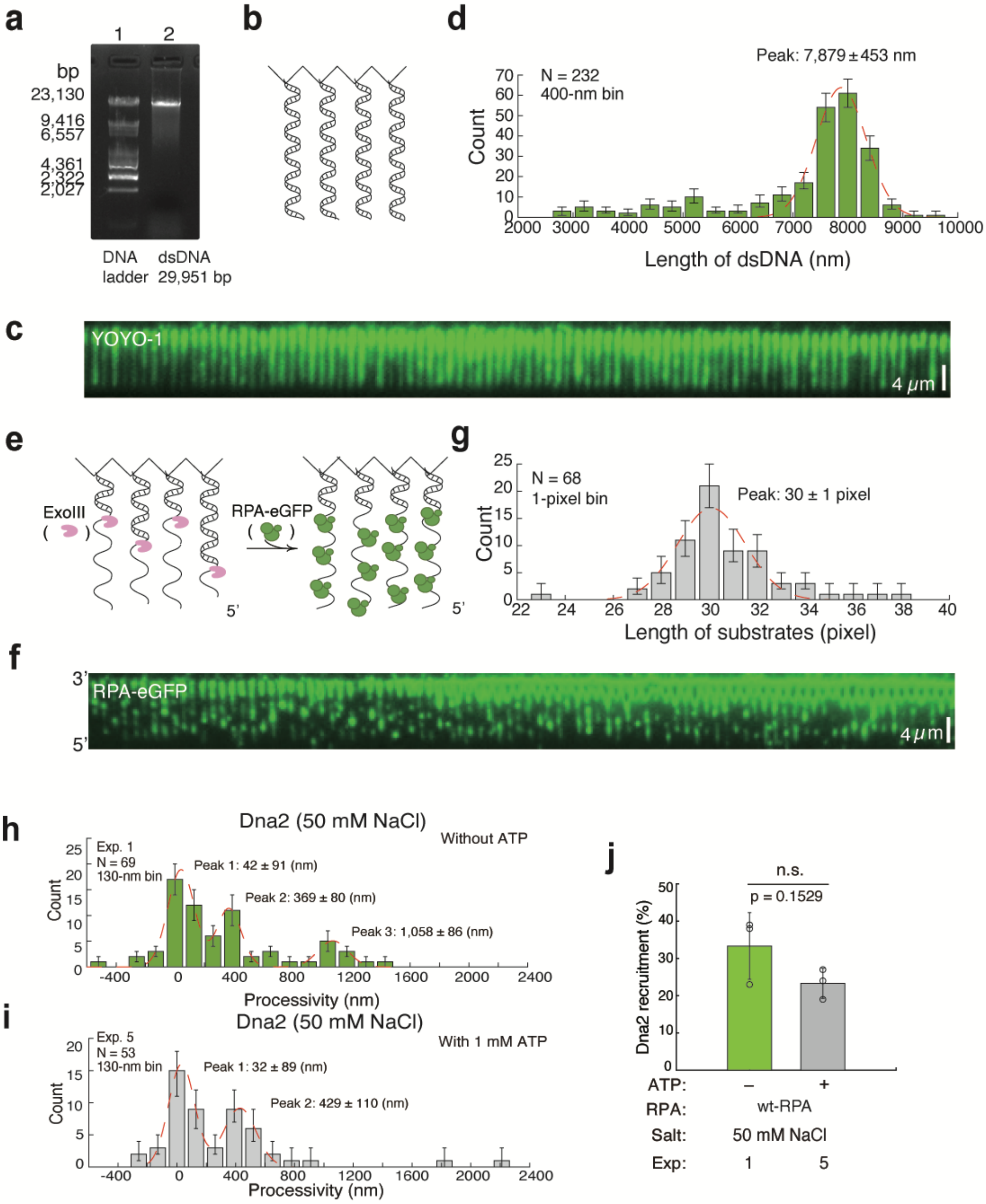

Supplementary Figure 2. DNA Curtains setup and impact of ATP on the processivity of Dna2-RPA ensemble. a. PCR product of the human β-globin dsDNA substrates (29,951-bp) and experiments were repeated three times. b-c. Schematic b and representative wide-field image c of DNA Curtains. DNA substrates were from a. d. Length distribution of DNA substrates in c. 400-nm bin. N = 232, which was the total number of DNA substrates examined over one time DNA Curtains experiment. e-f. Schematic e and representative wide-field image f DNA Curtains. DNA substrates were long 5'-ssDNA substrates (Methods). RPA-GFP was used to extend and image the substrates. g. Length distribution of ssDNA substrates in f. 1-pixel bin (1 pixel = 267 nm). h-i. Processivity distribution of single Dna2 digestion without (Exp. 1) and with 1mM ATP (Exp. 5) in solution (Exp.1 and Exp.5 in Supplementary Table 1). 130-nm bin. The total number of Dna2 digestion events: h. N = 69 for Exp. 1; i, N = 53 for Exp. 5. Each experimental condition was examined over more than three DNA Curtains experiments (n ≥ 3). j. Dna2 recruitment (%) with (Exp. 1) or without ATP (Exp. 5) in solution. Independent DNA Curtains experiments were repeated: n = 3 for Exp. 1; n = 3 for Exp.10; Error bars, mean ± s.d.. Error bars in d, g, h and i were obtained through the bootstrap analysis. For any normally distributed dataset 68.27% of the values lie within one standard deviation of the mean, therefore our choice of 70% confidence intervals for the bootstrapped data provides a close approximation to expectations for one standard deviation from the mean. The data were fitted with Gaussian functions (red dash line). The errors represented 95% confidence intervals obtained through Gaussian function fitting. Statistical significance in H(iii) was analyzed using the unpaired t-test for two groups. p-value: two-tailed; p-value style: GP: 0.1234 (ns), 0.0332 (*), 0.0021 (**), 0.0002 (***), <0.0001 (****). Confidence level: 95%. Source data are provided as a Source Data file.

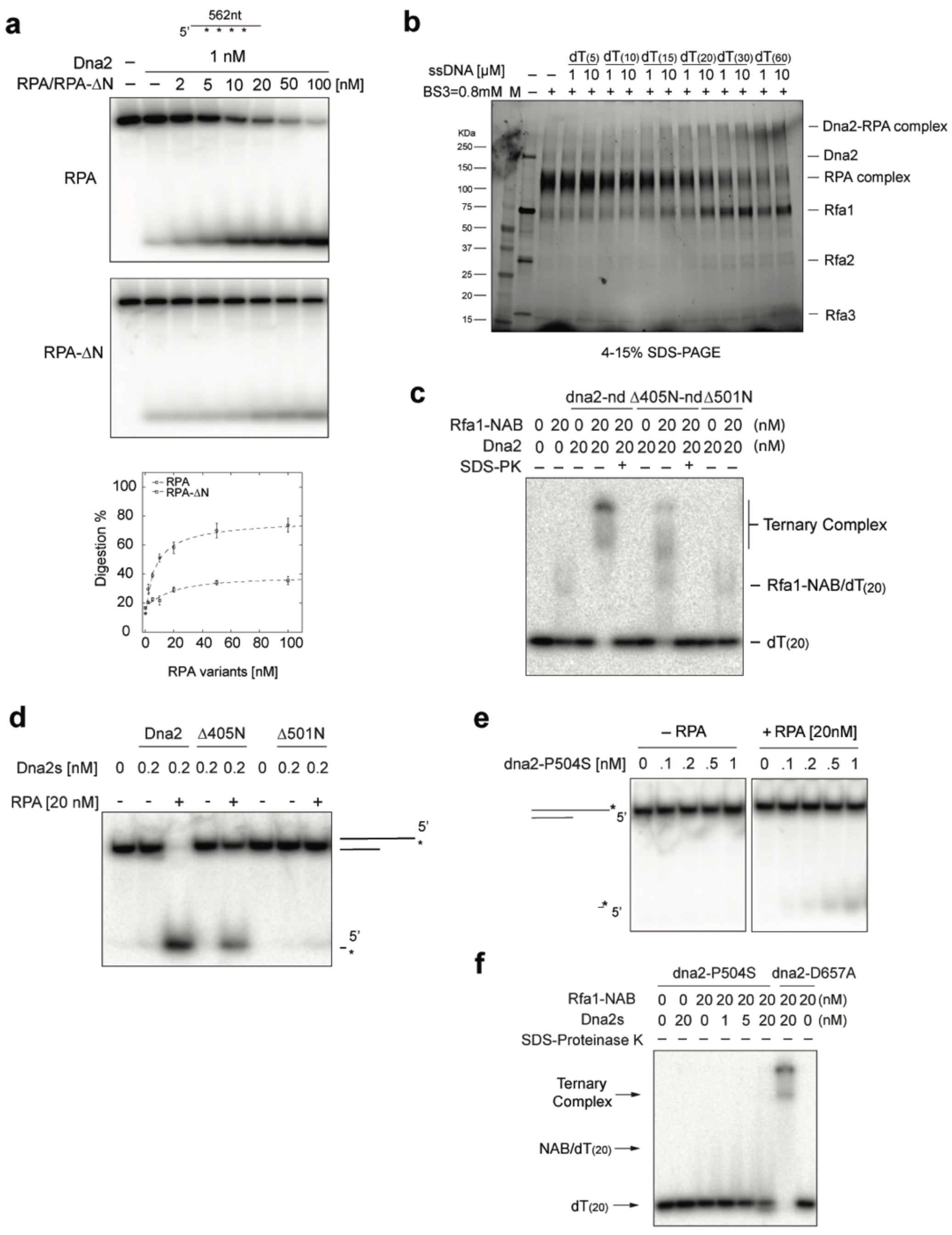

Supplementary Figure 3. Characterization of the Dna2-RPA-ssDNA ternary complex: the length of ssDNA and key regions from Dna2. a. Impact of titrated RPA and RPA-ΔN (0 nM to 100 nM) on the digestion of internally labeled 562-nt ssDNA (5 nM) by Dna2 (1 nM). For quantification, mean values ± s.d. from three independent experiments were plotted. b. Effect of the length of ssDNA (5, 10, 15, 20, 30 and 60 nt) on crosslinking efficiency of Dna2 (1 μM) and RPA (1 μM) and the experiments were carried out once. c. Formation of ternary complex from Dna2 variants (dna2-nd, dna2-Δ405N-nd and dna2-Δ501N) (20 nM), Rfa1-NAB (20 nM) and $dT_{(20)}$ ssDNA (5 nM). d. Digestion by Dna2, dna2-Δ405N and dna2-Δ501N (0.2 nM) on 5'-labeled 40-nt 5'-overhanging ssDNA (5 nM) without or with RPA (20 nM). e. Digestion by titrated dna2-P504S (0 nM to 1nM) on 5'-labeled 40-nt 5'-overhanging ssDNA (5 nM) without RPA or with RPA (20 nM). f. Failure of the formation of ternary complex from titrated dna2-P504S (0 nM to 20 nM), Rfa1-NAB (20 nM) and $dT_{(20)}$ ssDNA (5 nM). The experiments were repeated three times in c-f. Source data are provided as a Source Data file.

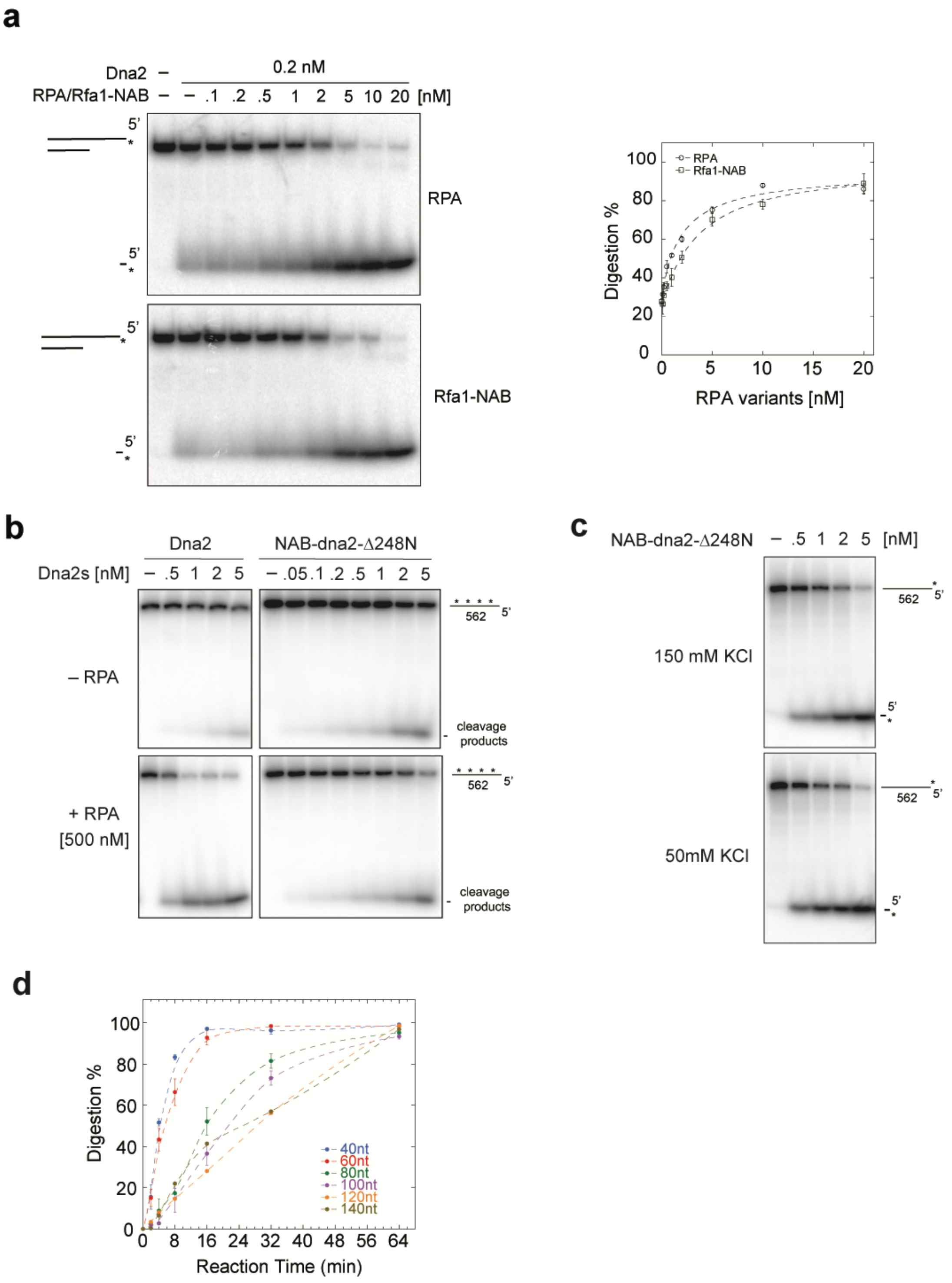

Supplementary Figure 4. Comparative characterization of the NAB-dna2Δ248N fusion and the Dna2-RPA complex. a. Comparison of titrated RPA and Rfa1-NAB (0 nM to 20 nM), on the digestion of 5'-labeled 40-nt 5'-overhanging ssDNA (5 nM) by Dna2 (0.2 nM). For quantification, mean values ± s.d. from three independent experiments were plotted. b. Digestion by titrated Dna2 and NAB-dna2Δ248N (0 nM to 5 nM) on internally labeled 562-nt ssDNA (5 nM) without or with RPA (500 nM). c. Digestion by titrated NAB-dna2Δ248N (0 nM to 5 nM) on 5'-labeled 562-nt ssDNA (5 nM) at 150 mM KCl and 50 mM KCl. The experiments were repeated three times in b-c. d. Time-course study of the impact of the length of ssDNA regions (40, 60, 80, 100, 120 and 140 nt) on the digestion of 3'-labeled 5'-overhanging ssDNA (5 nM) (hairpin removed) by NAB-dna2Δ248N (1 nM). Values from single experiment on the length of 120 and 140 nt, and the mean values ± s.d. from three independent experiments on the length of 40, 60, 80 and 100 nt were plotted. Source data are provided as a Source Data file.

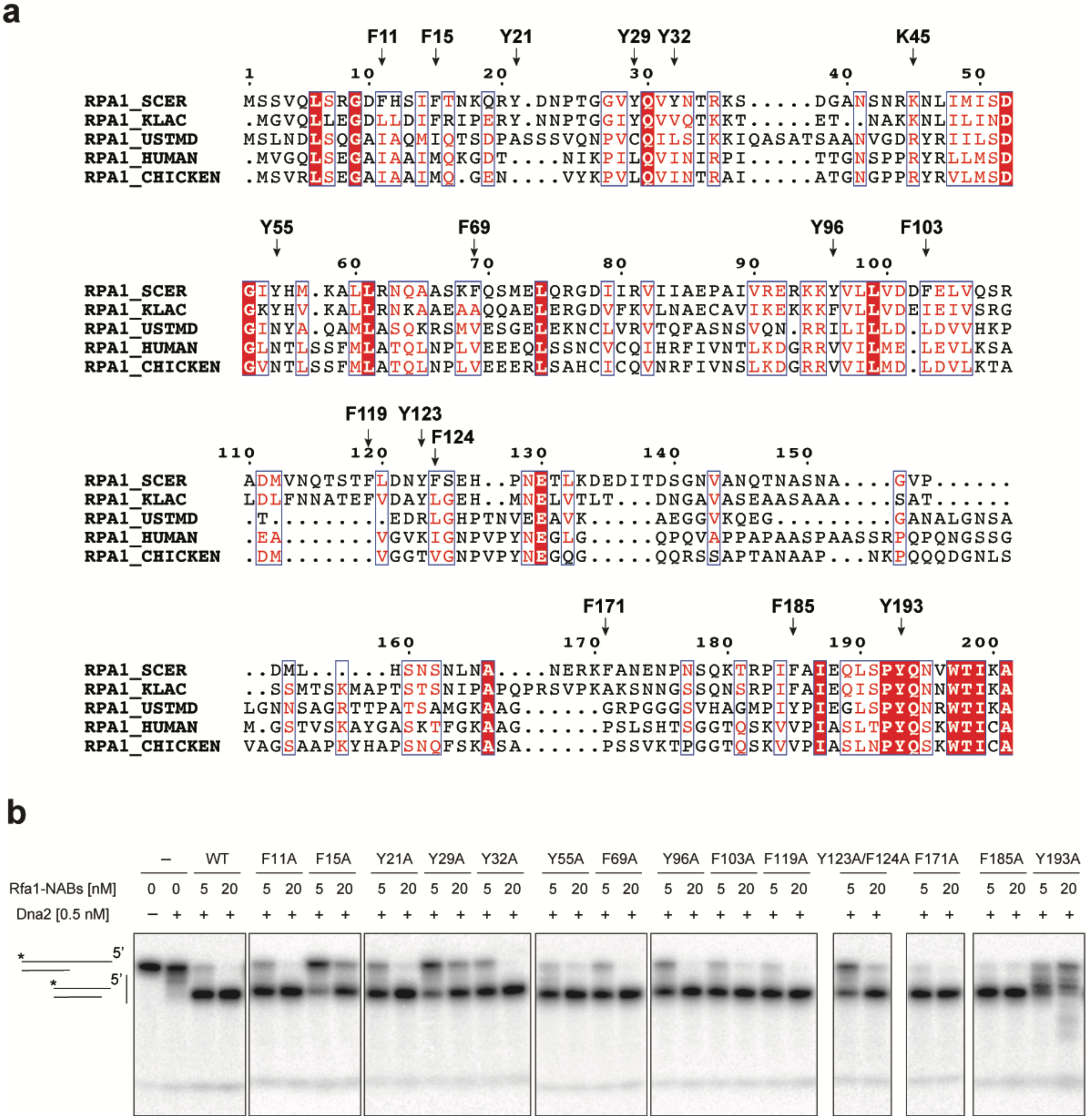

Supplementary Figure 5. Characterization of aromatic mutants from Rfa1-N. a. Protein sequence alignment of the N domain of RPA1 proteins across multiple eukaryotic species. (SCER: *Saccharomyces cerevisiae*; KLAC: *Kluyveromyces lactis*; USTMD: *Ustilago maydis*; HUMAN: *Homo sapiens*; CHICKEN: *Gallus gallus*). All aromatic residues of Rfa1-N from *S. cerevisiae* (F11, F15, Y21, Y29, Y32, Y55, F69, Y96, F103, F119, Y123, F124, F171, F185 and Y193A) and K45, were highlighted by black arrows. b. One-time screening digestion by Dna2 (0.5 nM) on 3'-labeled 40-nt 5'-overhanging ssDNA (5 nM) with wild-type Rfa1-NAB and Rfa1-NAB alanine mutants for aromatic residues from Rfa1-N (5 nM and 20 nM): F11A, F15A, Y21A, Y29A, Y32A, Y55A, F69A, Y96A, F103A, F119A, Y123A/F124A, F171A, F185A and Y193A.

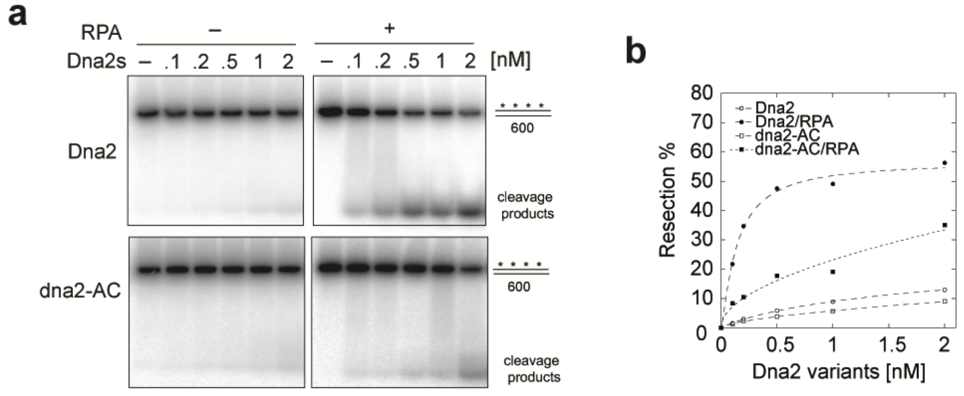

Supplementary Figure 6. dna2-AC is defective in DNA end resection in vitro. a. Resection of internally labeled 600-nt dsDNA (5 nM) by titrated Dna2 and dna2-AC (0 nM to 2 nM) in the presence of Sgs1 (10 nM) and Top3-Rmi1 (10 nM) complex, without and with RPA (500 nM) and the experiments were carried out once. b. The results from one-time experiments were quantified and plotted. Source data are provided as a Source Data file.

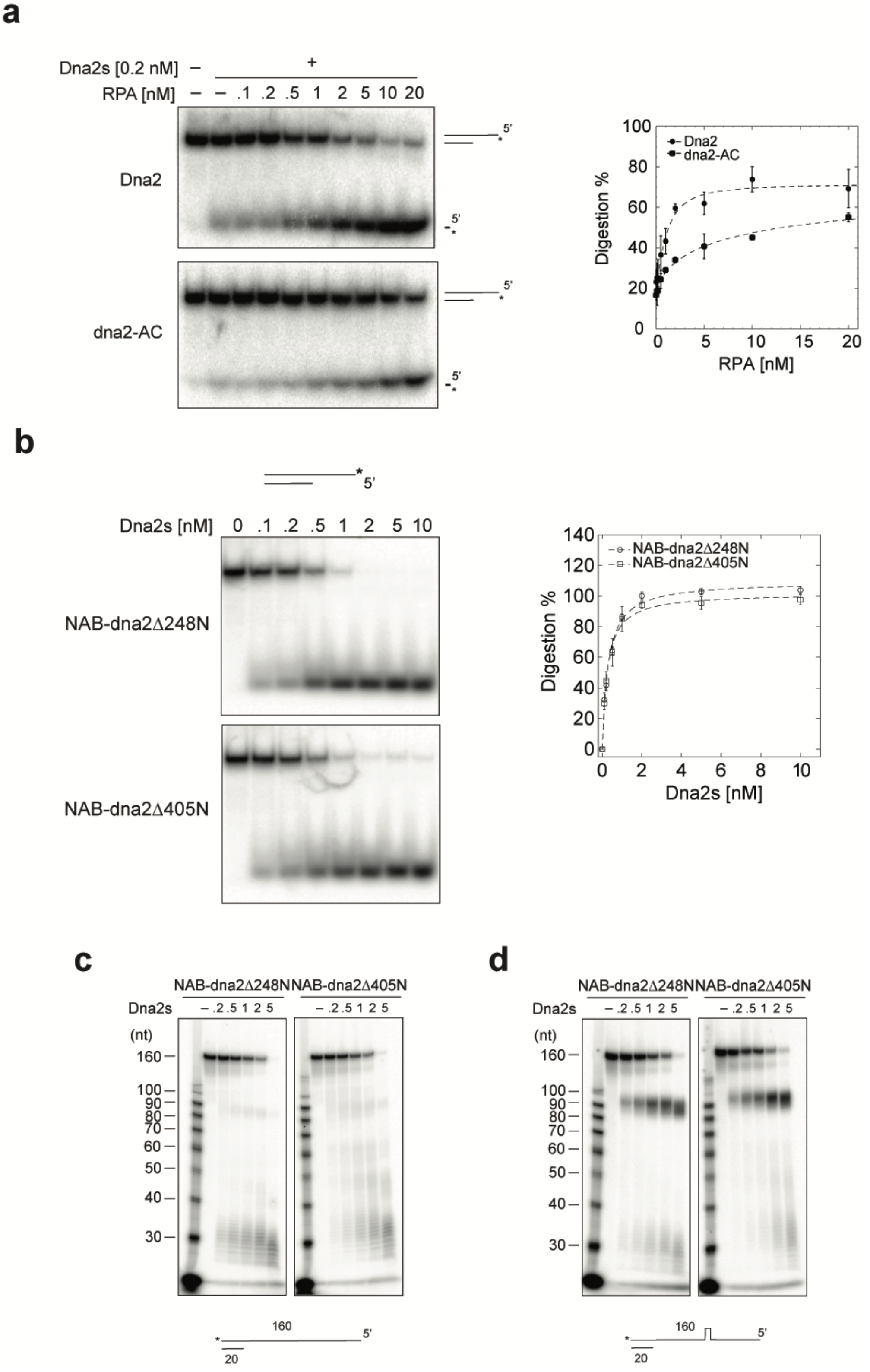

Supplementary Figure 7. Nuclease assays of dna2-AC and NAB-dna2-Δ405N. a. Impact of titrated RPA (0 nM to 20 nM) on the digestion of 5'-labeled 40-nt overhanging ssDNA (5 nM) by Dna2 and dna2-AC (0.2 nM). For quantification, mean values ± s.d. from three independent experiments were plotted. b. Comparison of titrated NAB-dna2Δ248N and NAB-dna2Δ405N (0 nM to 10 nM), on the digestion of 5'-labeled 40-nt overhanging ssDNA (5 nM). For quantification, mean values ± s.d. from three independent experiments were plotted. c-d. Comparison of titrated NAB-dna2Δ248N and NAB-dna2Δ405N (0 nM to 10 nM), on the digestion of 3'-labeled 140-nt 5'-overhanging ssDNA (5 nM) (hairpin-removed) (c) and 3'-labeled 140-nt 5'-overhanging ssDNA (5 nM) (hairpin-containing) (d). The experiments were repeated three times in c-d. Source data are provided as a Source Data file.

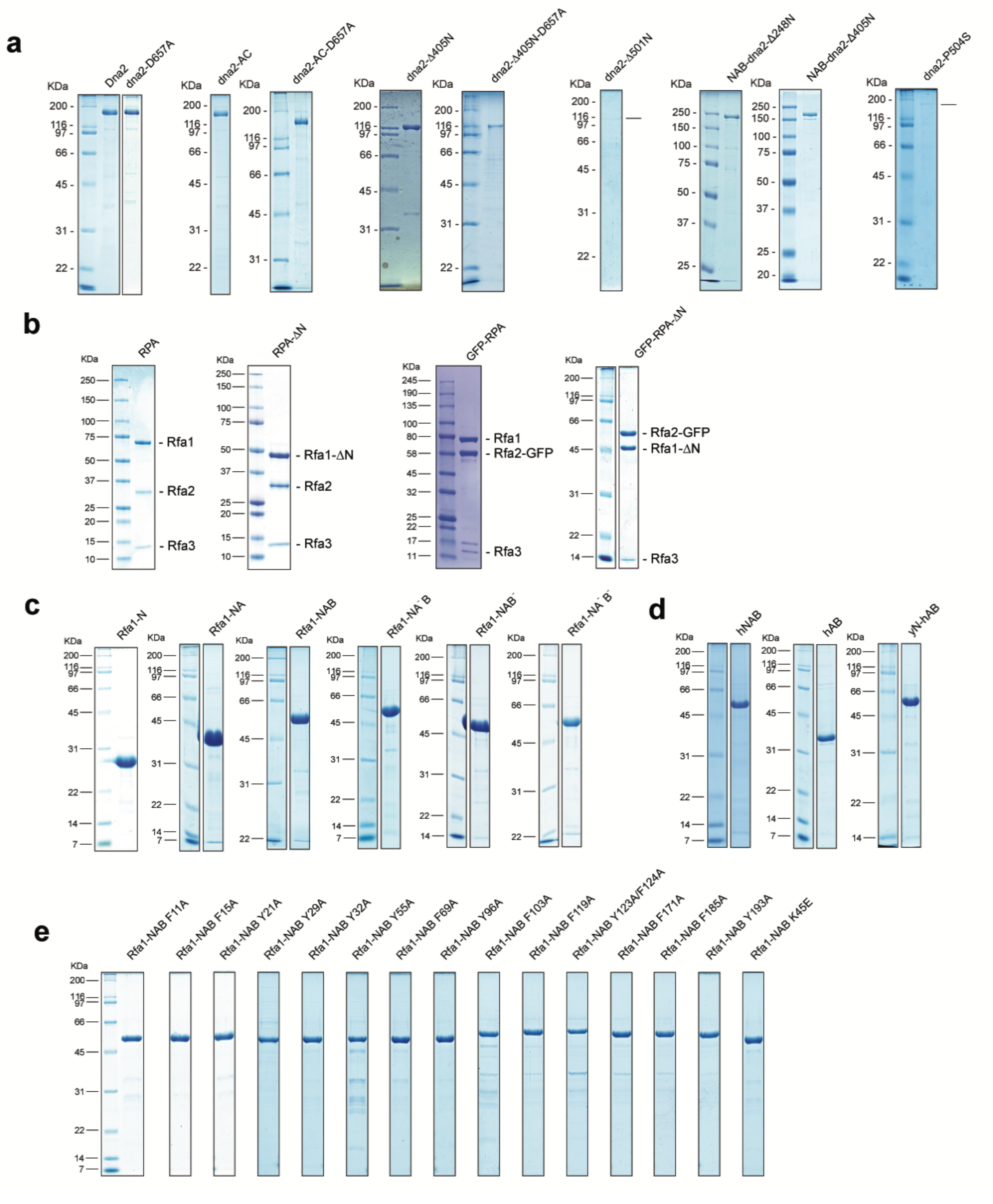

Supplementary Figure 8. SDS-PAGE analyses of purified proteins. a. Purified Dna2 and dna2-D657A proteins, dna2-AC and dna2-AC-D657A proteins, dna2-Δ405N and dna2-Δ405N-D657A proteins, dna2-Δ501N protein, NAB-dna2-Δ248N and NAB-dna2-Δ405N proteins, and dna2-P504S protein, were analyzed by 10% SDS-PAGE. b. Purified RPA and RPA-ΔN proteins, GFP-RPA and GFP-RPA-ΔN proteins, were analyzed by 4-15% gradient or 12.5% SDS-PAGE. c. Purified Rfa1-N, Rfa1-NA, Rfa1-NAB, Rfa1-NA$^-$B, Rfa1-NAB$^-$ and Rfa1-NA$^-$B$^-$ proteins were analyzed by 12.5% SDS-PAGE. d. Purified hNAB, hAB and NhAB proteins were analyzed by 12.5% SDS-PAGE. e. Purified Rfa1-NAB F11A, F15A, Y21A, Y29A, Y32A, Y55A, F69A, Y96A, F103A, F119A, Y123A/Y124A, F171A, F185A, F193A and K45E proteins were analyzed by 12.5% SDS-PAGE. The experiments were carried out once in a-e.

Supplementary Tables

Supplementary Table 1. DNA Curtains experiments (Methods)

#	Experimental conditions
Exp. 1	2 nM Dna2 with a working buffer containing 0.1 nM RPA-GFP and 50 mM NaCl
Exp. 2	2 nM Dna2 with a working buffer containing 0.1 nM RPA-GFP and 150 mM NaCl
Exp. 3	2 nM Dna2-D657A with a working buffer containing 0.1 nM RPA-GFP and 50 mM NaCl
Exp. 4	2 nM Dna2 with a working buffer containing no RPA-GFP and 50 mM NaCl
Exp. 5	2 nM Dna2 with a working buffer containing 0.1 nM RPA-GFP, 50 mM NaCl and 1 mM ATP
Exp. 6	2 nM Dna2 pre-incubated with 2 nM RPA-GFP, with a working buffer containing no RPA-ΔN-GFP and 50 mM NaCl
Exp. 7	2 nM Dna2 with a working buffer containing 0.1 nM RPA-Y193A-GFP and 150 mM NaCl
Exp. 8	2 nM Dna2-AC with a working buffer containing 0.1 nM RPA-GFP and 50 mM NaCl
Exp. 9	2 nM Dna2-AC with a working buffer containing 0.1 nM RPA-GFP and 150 mM NaCl
Exp. 10	2 nM Dna2-AC with a working buffer containing no RPA-GFP and 150 mM NaCl

Note: The working buffer for DNA Curtains was 20 mM Tris-HCl (pH 7.5), 2 mM $MgCl_2$, 1 mM DTT, 50/150 mM NaCl, and 0.2 mg/ml BSA.

Supplemental Table 2. Crosslinked sites within the Dna2-RPA-ssDNA ternary complex identified by mass spectrometry.

Protein 1	Protein 2	Protein 1 XLink AA	Protein 2 XLink AA	Rank*
Dna2	Dna2	9	20	3
Dna2	Dna2	20	25	1
Dna2	Dna2	20	41	3
Dna2	Dna2	68	82	2
Dna2	Dna2	82	90	1
Dna2	Dna2	246	282	1
Dna2	Dna2	411	447	2
Dna2	Dna2	411	471	1
Dna2	Dna2	411	1498	1
Dna2	Dna2	1128	1498	2
Dna2	Rfa1	9	0	3
Dna2	Rfa1	20	0	1
Dna2	Rfa1	82	0	1
Dna2	Rfa1	246	0	1
Dna2	Rfa1	282	0	1
Dna2	Rfa1	346	0	2
Dna2	Rfa1	347	0	1
Dna2	Rfa1	411	0	1
Dna2	Rfa1	434	0	1
Dna2	Rfa1	488	0	1
Dna2	Rfa1	488	170	2
RFA1	Rfa1	0	170	1
RFA1	Rfa1	36	170	1
RFA1	Rfa1	259	170	2
Rfa2	Rfa3	146	0	2

*Rank 1: high confidence group, data included cross-linked di-peptides for which the lower scoring peptide had at least three unique y-type or b-type fragment ions identified.

*Rank 2: low confidence group, data included cross-linked di-peptides for which the lower scoring peptide had only one or two unique y-type or b-type fragment ions identified.

*Rank 3: mass-alone group, data included cross-linked di-peptides for which the lower scoring peptide had less than six amino acid residues and thus had a much-reduced chance of identifying three unique fragment ions.

Supplementary Table 3. Yeast strains and genotypes.

Strain name	Parental Strain	Genotype	Source
JKM139		*MATa ho hml::ADE1 hmr::ADE1 ade1 leu2-3,-112 lys5 trp1::hisG ura3-52 lys5 ade3::GAL10::HO*	[1]
JKM179		*MATα ho hml::ADE1 hmr::ADE1 ade1 leu2-3,-112 lys5 trp1::hisG ura3-52 lys5 ade3::GAL10::HO*	[1]
yWH361	JKM139	*pif1-m2*	[2]
yWH475	yWH361	*pif1-m2 dna2::KANMX*	[2]
NP148	yWH361	*pif1-m2 dna2-AC*	This study
NP196	JKM139	*dna2-AC*	This study
NP216	JKM139	*exo1::KANMX*	This study
NP212	JKM139	*exo1::KANMX dna2-AC*	This study

References:

1 Lee, S. E. *et al. Saccharomyces* Ku70, Mre11/Rad50 and RPA proteins regulate adaptation to G2/M arrest after DNA damage. *Cell* **94**, 399-409, doi:10.1016/s0092-8674(00)81482-8 (1998).

2 Zhu, Z., Chung, W. H., Shim, E. Y., Lee, S. E. & Ira, G. Sgs1 helicase and two nucleases Dna2 and Exo1 resect DNA double-strand break ends. *Cell* **134**, 981-994, doi:10.1016/j.cell.2008.08.037 (2008).

Supplementary Table 4. The sequence of oligonucleotides used in biochemical assays.

Name	Sequence
S1	CATATTTAAAACATGTTGGATCCCAGCACCAGATTCAGCA
S2	TTGATAAGAGGTCATTTGAATTCATGGCTTAGAGCTTAATTGCTGAATCTGGTGC TGGGATCCAACATGTTTTAAATATG
S3	TGTCGGACTTACGTATTATGTTGATAAGAGGTCATTTGAATTCATGGCTTAGAGC TTAATTGCTGAATCTGGTGCTGGGATCCAACATGTTTTAAATATG
S4	CATGCCATGGGCAGCAGTGTTCAACTTTCGAGGGGCGATTTTCATAGCATCTTCA CCAATAAGCAAAGGTATTTTTTTCCCACCGGTGGCGTTTATCAAGTTTATAACAC CAGGAAATCTGATGGGGCTAACAGCAACAGAAAGAATTTGATCATGATTT
S5	AAATCATGATCAAATTCTTTCTGTTGCTGTTAGCCCCATCAGATTTCCTGGTGTTA TAAACTTGATAAACGCCACCGGTG
S6	AAATCATGATCAAATTCTTTCTGTTGCTGTTAGCCCCATCAGATTTCCTGGTGTTA TAAA
S7	AAATCATGATCAAATTCTTTCTGTTGCTGTTAGCCCCATC
S8	AAATCATGATCAAATTCTTT
S9	CATGCCATGGGCAGCAGTGTTCAACTTTCGAGGGGCGATTTTCATAGCATCTTCA CCAATAAGCAAAGGTACGATAATCCCACCGGTGGCGTTTATCAAGTTTATAACA CCAGGAAATCTGATGGGGCTAACAGCAACAGAAAGAATTTGATCATGATTT
S10	TTTTTTTTTTTTTTTTTTTTTTTTTTTTTTTTGATAAGAGGTCATTTGAA
S11	TTCAAATGACCTCTTATCAA
S12	TTCATGGCTTAGAGCTTAATTTTTTTTTTTTTTTTTTTTTTTTTTTTTTT
S13	ATTAAGCTCTAAGCCATGAA
S14	TTCATGGCTTAGAGCTTAATTTTTTTTTTTTTTTTTTTTTTTTTTTTTTTTTGATAAG AGGTCATTTGAA
