# Supplementary material for: Deciphering the mechanism of processive ssDNA digestion by the Dna2-RPA ensemble
Source: Nat Commun. 2022 Jan 18;13:359. doi: 10.1038/s41467-021-27940-y (PMC8766458; doi:10.1038/s41467-021-27940-y)
Supplement: Supplementary file 3 — Description of Additional Supplementary Files [file 41467_2021_27940_MOESM3_ESM.pdf]

Description of Additional Supplementary Files

File Name: Supplementary Movie 1
Description: Single molecule of Dna2 digestion on RPAssDNA complex. QD labeled single Dna2 digested on individual RPAGFP coated ssDNA molecules with a free 5‟ end (Fig. 1B).
