# Supplementary material for: Deciphering the mechanism of processive ssDNA digestion by the Dna2-RPA ensemble
Source: Nat Commun. 2022 Jan 18;13:359. doi: 10.1038/s41467-021-27940-y (PMC8766458; doi:10.1038/s41467-021-27940-y)
Supplement: Supplementary file 5 — Reporting Summary [file 41467_2021_27940_MOESM5_ESM.pdf]

nature portfolio

Corresponding author(s): Hengyao Niu and Zhi Qi

Last updated by author(s): Nov 20, 2021

Reporting Summary

Nature Portfolio wishes to improve the reproducibility of the work that we publish. This form provides structure for consistency and transparency in reporting. For further information on Nature Portfolio policies, see our Editorial Policies and the Editorial Policy Checklist.

Statistics

For all statistical analyses, confirm that the following items are present in the figure legend, table legend, main text, or Methods section.

n/a	Confirmed	
☐	☒	The exact sample size (*n*) for each experimental group/condition, given as a discrete number and unit of measurement
☐	☒	A statement on whether measurements were taken from distinct samples or whether the same sample was measured repeatedly
☐	☒	The statistical test(s) used AND whether they are one- or two-sided<br>*Only common tests should be described solely by name; describe more complex techniques in the Methods section.*
☒	☐	A description of all covariates tested
☐	☒	A description of any assumptions or corrections, such as tests of normality and adjustment for multiple comparisons
☐	☒	A full description of the statistical parameters including central tendency (e.g. means) or other basic estimates (e.g. regression coefficient) AND variation (e.g. standard deviation) or associated estimates of uncertainty (e.g. confidence intervals)
☐	☒	For null hypothesis testing, the test statistic (e.g. *F*, *t*, *r*) with confidence intervals, effect sizes, degrees of freedom and *P* value noted<br>*Give P values as exact values whenever suitable.*
☒	☐	For Bayesian analysis, information on the choice of priors and Markov chain Monte Carlo settings
☒	☐	For hierarchical and complex designs, identification of the appropriate level for tests and full reporting of outcomes
☐	☒	Estimates of effect sizes (e.g. Cohen's *d*, Pearson's *r*), indicating how they were calculated

Our web collection on statistics for biologists contains articles on many of the points above.

Software and code

Policy information about availability of computer code

Data collection	1. Phosphor-imaging data were collected by scanning with Typhoon 5 Biomolecular Imager controlled by ImageQuant TL 8 software from GE Health Care.<br>2. DNA Curtains data were acquired with a custom-built prism-type total internal reflection fluorescence microscope (TIRFM) (Nikon, Inverted Microscope Eclipse Ti-E). The software was CellVision Coolight Technology Version 1.4.0 (Home-made software).<br>3. Tryptic digestion of cross-linked di-peptides was analyzed by an Easy-nLC 1000 HPLC system coupled to an Orbitrap Fusion Lumos mass spectrometer from Thermo Scientific. Thermo Scientific Xcalibur software version 4.1 was used to control and acquire mass spectrometry data from LC and MS instruments.<br>4. Microscale thermophoresis data were collected by Monolith NT.115 Microscale Thermophoresis device controlled by NT Control software, from Nano Temper Technologies.
Data analysis	1. Phosphor-imaging data collected from Typhoon scanner was analyzed and quantified using ImageJ 1.53i, and graphed in Kaleidagraph v4.5 and Graphpad Prism 9.3.0. .<br>2. Confocal microscopy data were collected and analyzed by Nikon Elements.<br>3. Consecutive motif and total motif analysis was conducted by MATLAB 2016b software (https://www.mathworks.com/products/matlab.html).<br>4. Microscale thermophoresis data was analyzed and binding constant Kd was determined in Kd Fit mode in NT Analysis software.

For manuscripts utilizing custom algorithms or software that are central to the research but not yet described in published literature, software must be made available to editors and reviewers. We strongly encourage code deposition in a community repository (e.g. GitHub). See the Nature Portfolio guidelines for submitting code & software for further information.

Data

Policy information about availability of data

All manuscripts must include a data availability statement. This statement should provide the following information, where applicable:

- Accession codes, unique identifiers, or web links for publicly available datasets
- A description of any restrictions on data availability
- For clinical datasets or third party data, please ensure that the statement adheres to our policy

1. The resulting mass spectrometry data was searched in Protein Prospector v5.24.1 (http://prospector.ucsf.edu/prospector/mshome.htm) and the final mass spectrometry proteomics data have been deposited to the ProteomeXchange Consortium via the PRIDEpartner repository with the dataset identifier PXD028637.
2. The data that support the findings of this study are available from the corresponding author upon reasonable request. The source data underlying Figs 1-8, and Supplementary Figs 1-8 are provided as a Source Data file.

Field-specific reporting

Please select the one below that is the best fit for your research. If you are not sure, read the appropriate sections before making your selection.

- [x] Life sciences
- [ ] Behavioural & social sciences
- [ ] Ecological, evolutionary & environmental sciences

For a reference copy of the document with all sections, see nature.com/documents/nr-reporting-summary-flat.pdf

Life sciences study design

All studies must disclose on these points even when the disclosure is negative.

Sample size	For resection analysis, at least 50 million cells were collected for each mutants per time point. For drug sensitivity assay, 3000 - 5000 cells were collected before dilution to spot on drug-supplemented media plate. For biochemical experiments, results from three independent experiments were used to calculate the mean value and the standard deviation. The choices of the sample sizes are based on mature protocols established in the field of research and have been proved to be reliable.
Data exclusions	No data were excluded in this study.
Replication	All biochemical experiments in this study, including nuclease assays, resection assays, EMSA assays, pull-down assays and MST assays, were repeated equal or more than three times. All high-throughput DNA Curtains experiments were repeated three times. All genetic experiments in this study were repeated three times. BS3-crosslinking experiments were performed and submit to mass-spectrometry analysis once. All attempts at replication were successful, except when technical errors happened to prevent to obtain the final results.
Randomization	All experiments involve randomization.
Blinding	Not applicable in this study because experimental targets are typically purified protein and nucleic acid molecules that are biochemically highly homogenized and do not tend to display individual diverse properties. For genetic studies using yeast cells, each individual yeast cell's "expectation" does not have an impact on the research results. Thus, we chose not to perform any blinding experiment in this manuscript.

Reporting for specific materials, systems and methods

We require information from authors about some types of materials, experimental systems and methods used in many studies. Here, indicate whether each material, system or method listed is relevant to your study. If you are not sure if a list item applies to your research, read the appropriate section before selecting a response.

Materials & experimental systems

n/a	Involved in the study	
[ ]	[x]	Antibodies
[ ]	[x]	Eukaryotic cell lines
[x]	[ ]	Palaeontology and archaeology
[x]	[ ]	Animals and other organisms
[x]	[ ]	Human research participants
[x]	[ ]	Clinical data
[x]	[ ]	Dual use research of concern

Methods

n/a	Involved in the study	
[x]	[ ]	ChIP-seq
[x]	[ ]	Flow cytometry
[x]	[ ]	MRI-based neuroimaging

Antibodies

Antibodies used	Monoclonal ANTI-FLAG BioM2 antibody (Sigma F9291-.2mg) was used to prepare anti-FLAG tagged quantum dots. 1.0 pmole anti-

	Flag QDs were pre-incubated with 0.2 pmole Dna2 proteins containing a Flag tag.
Validation	The antibody used here was conjugated to DIBO-modified Qdot705 in order to label the proteins with FLAG-tag. 0.125mg antibody was used in the antibody labeling kit (Life technologies, Cat. MP10469). For a previous successful example please refer to: Sternberg, S. H., Redding, S., Jinek, M., Greene, E. C. & Doudna, J. A. DNA interrogation by the CRISPR RNA-guided endonuclease Cas9. Nature 507, 62-67, doi:10.1038/nature13011 (2014).

Eukaryotic cell lines

Policy information about cell lines

Cell line source(s)	Saccharomyces cerevisiae strains were constructed from frozen stocks of JKM139 and JKM179 reported before
Authentication	Mating type and marker genotype were authenticated by growth on selective plates.
Mycoplasma contamination	Yeast are not susceptible to mycoplasma contamination.
Commonly misidentified lines (See ICLAC register)	No commonly misidentified cell lines were used.
