# Supplementary material for: Deciphering the mechanism of processive ssDNA digestion by the Dna2-RPA ensemble
Source: Nat Commun. 2022 Jan 18;13:359. doi: 10.1038/s41467-021-27940-y (PMC8766458; doi:10.1038/s41467-021-27940-y)

Southern blot Figure 6: dotted rectangles correspond to samples included in the figure.

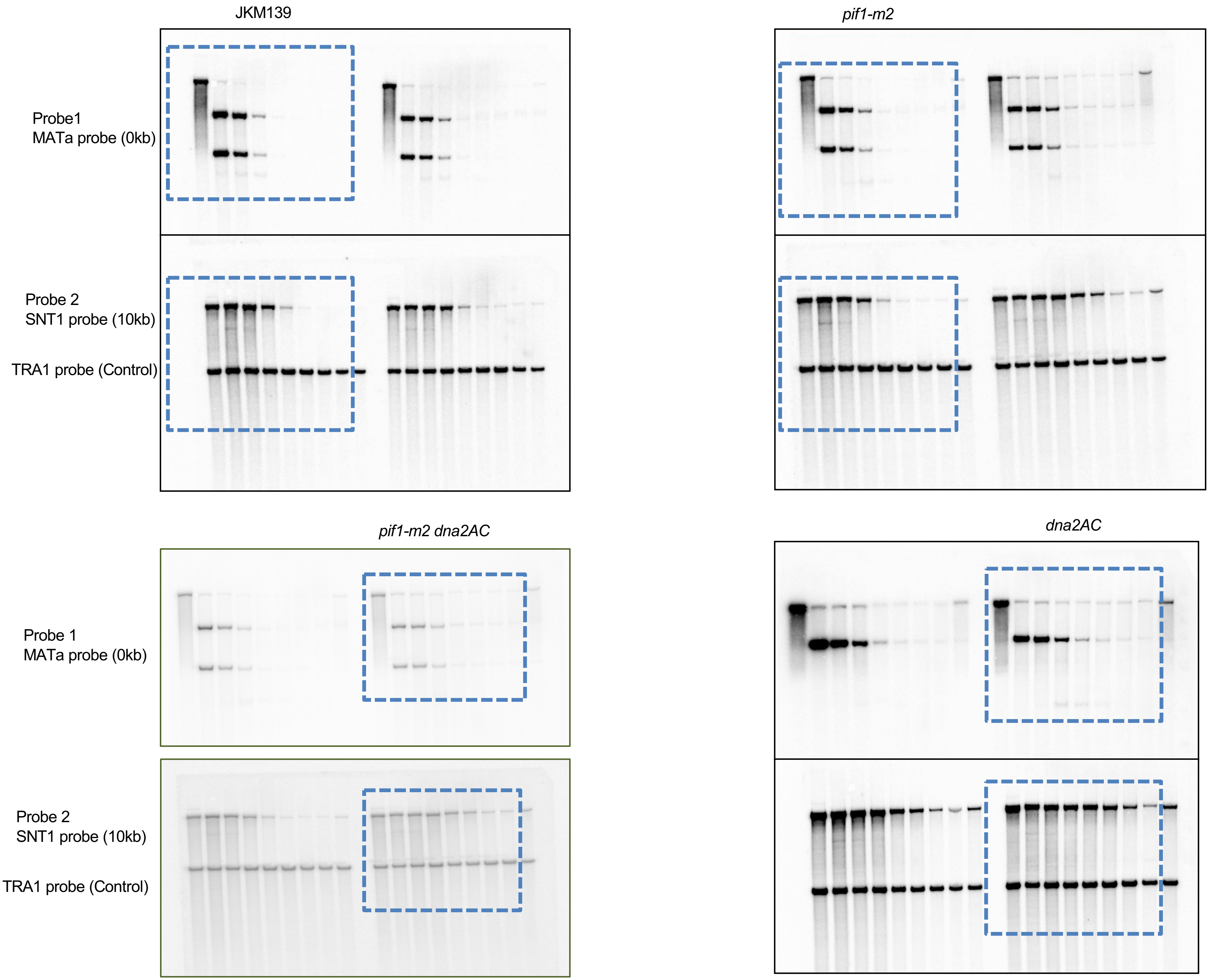

exo1

Probe 1
MATa probe (0kb)

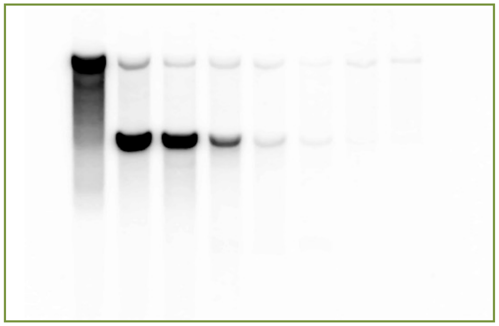

Probe 2
SNT1 probe (10kb)

TRA1 probe (Control)

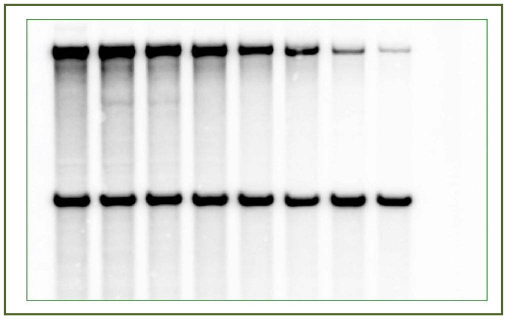

dna2AC exo1

Probe 1
MATa probe (0kb)

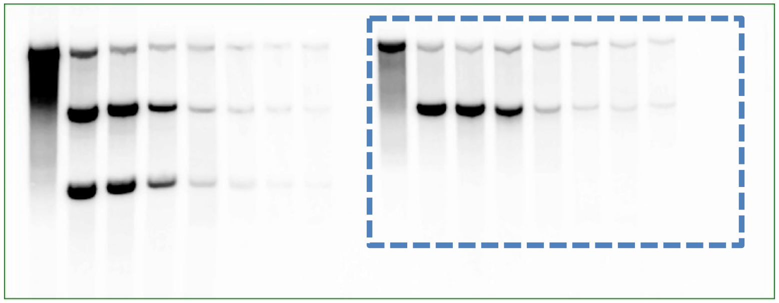

Probe 2
SNT1 probe (10kb)

TRA1 probe (Control)

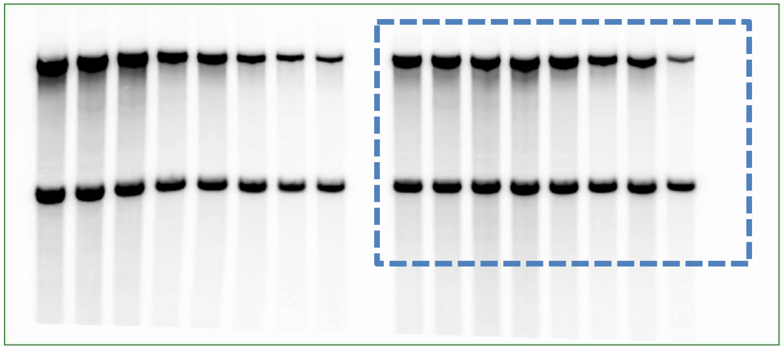

pif1-m2 dna2

Probe 1
MATa probe (0kb)

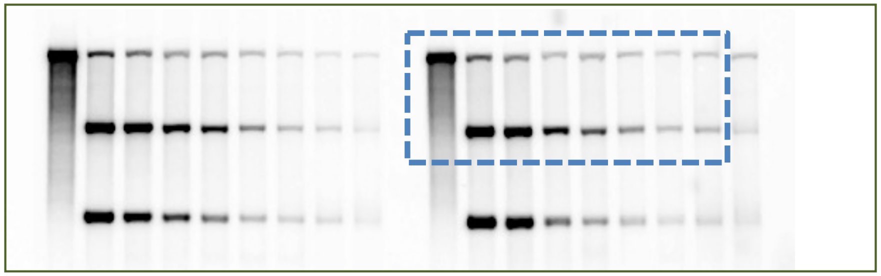

Probe 2
SNT1 probe (10kb)

TRA1 probe (Control)

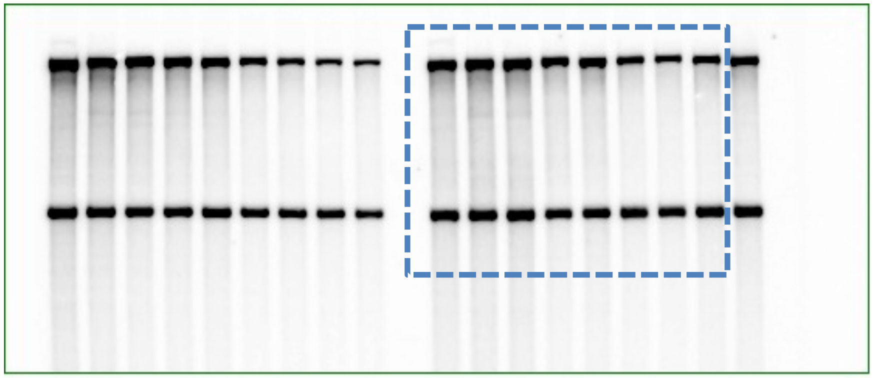

Supplement: Supplementary file 6 — Source Data [file 41467_2021_27940_MOESM6_ESM.zip › Uncropped blot for Fig. 6.pdf]
